# Hematological Changes in Opium Addicted Diabetic Rats

**DOI:** 10.5812/ijhrba.8777

**Published:** 2013-03-12

**Authors:** Gholamreza Asadikaram, Majid Sirati-Sabet, Majid Asiabanha, Nader Shahrokhi, Abdollah Jafarzadeh, Mohammad Khaksari

**Affiliations:** 1 Department of Biochemistry, Faculty of Medicine, Kerman University of Medical Sciences, Kerman, IR Iran; 2 Physiology Research Center, Kerman University of Medical Sciences, Kerman, IR Iran; 3 Department of Biochemistry, School of Medicine, Qazvin University of Medical Sciences, Qazvin, IR Iran; 4 Department of Physiology, Kerman University of Medical Sciences, Kerman, IR Iran; 5 Department of Immunology, Rafsanjan University of Medical Sciences, Kerman, IR Iran

**Keywords:** Drug Addiction, Diabetes Mellitus, Opium, Blood Cell Count, Hematological

## Abstract

**Background:**

Chronic opioid treatment in animal models has shown to alter hematological parameters.

**Objectives:**

The aim of this study was to evaluate the biological effects of opium on the number of peripheral blood cells and red blood cells (RBCs) indices in diabetic rats.

**Materials and Methods:**

Peripheral blood samples were collected from diabetic, opium-addicted, diabetic opium-addicted and normal male and female rats and hematological parameters were measured.

**Results:**

The mean number of white blood cells (WBCs) was significantly higher in diabetic opium-addict females compared to diabetic non-addict female group. In both male and female, the mean number of neutrophils was significantly higher and the mean number of lymphocytes was lower in diabetic opium-addicted rats than those observed in diabetic non-addicted group. In diabetic opium-addicted male group the mean counts of RBC significantly increased as compared with diabetic male group. However, in diabetic addicted female, the mean number of RBCs was significantly lower than diabetic non-addicted female group. In both males and females, the mean number of platelets was significantly lower in diabetic addict rats compared to diabetic non-addict group.

**Conclusions:**

Generally, the results indicated that opium addiction has different effects on male and female rats according to the number of WBC, RBC and RBC indices. It could also be concluded that in the opium-addicts the risk of infection is enhanced due to the weakness of immune system as a result of the imbalance effect of opium on the immune cells.

## 1. Background

Opioid drugs are the main substances of drug abuse and abusing opiate still remains a worldwide social problem (1). Opium is used as the raw material for the synthesis of some alkaloids including morphine, noscapine, papaverine and codeine which contain 8-17 %, 1-10 %, 0.5-1.5 % and 0.7-5 % of opium, respectively (2). Accumulating data indicated that opioid receptors, which are expressed by immune cells, are mainly similar to neuronal type opioid receptors, particularly k and δ-opioid type receptors. Researchers reported the presence of novel specific opioid receptors for morphine on lymphocytes. It is suggested that opioid receptor axis functions in an autocrine or paracrine manner (3). Inhibition of several leukocyte functions such as phagocytosis, chemotactic response, cytokine production, and generation of reactive oxygen intermediates and arachidonic acid derivatives have all been reported following the treatment with morphine. The variation between in-vivo and in-vitro data suggest that effects of morphine on some immune cells appear after in-vivo morphine abuse are probably not direct (4). Morphine can directly depress the function of macrophages and polymorphonuclear leukocytes (PMNs), and regulate expression of one type of T-cell surface marker. Opioid alkaloids and peptides such as morphine and the endogenous opioid peptides, including β-endorphin and the dynorphin peptides, modulate the function of lymphocytes and other cells involved in host defense and immunity (5).

Most of the reported data bases have suggested that opiates are involved in the regulation of cell-mediated immune responses in heroin addicts (6). It has also been reported that a single injection of heroin produces a dose dependent, naltrexone reversible decrease in the total number of leukocytes in the rat spleen. The heroin mediated decrease in the number of splenic leukocytes is not associated with a heroin-induced increase in circulating leukocytes. Heroin does not increase the number of necrotic leukocytes in the spleen, but it increases the number of apoptotic leukocytes in the spleen (7). It has been demonstrated that noscapine has potent antitumor activity without inhibition of immune responses (8). Moreover, noscapine decreases proliferation of cells at cell cultures (9). It has been demonstrated that NOS can induce chromosomal loss, hyperdiploidy and hypodiploidy in cultured human lymphocytes (10). These effects can directly interfere with lymphocytes function.

Papaverine, another opium alkaloids, could damage endothelial and smooth muscle cells by inducing changes associated with events leading to apoptosis (11). No investigation has been conducted for the evaluation of the effects of papaverine and noscapine on immune system to date. However, it was shown that papaverine induces concentration of dependent inhibition of the absorption of adenosine by thymocytes and also it was found that nucleoside transport into lymphocyte inhibited by papaverine (12, 13). These phenomena can influence lymphocytes metabolism and function .It has been also demonstrated that papaverine has potential effects on inducing a highly fatal syndrome as constrictive bronchiolitis obliterans. This capability of papaverine has been associated with the production of transforming growth factor-β (TGF-β) which has potent immunosuppression effects (14). There are, however, more than 20 alkaloids (15) and more than 70 components (16) in opium, thus, its effect on cell functions could be different from pure morphine, noscapine, codeine and papaverine.

Our previous findings on the effects of opium on biochemical parameters (17, 18), TGF-β (19), apoptosis (20) and also the influence of opium components on cell functions encouraged us to examine the biological effects of opium on peripheral blood cells of addicted rats. Due to the fact that some people around the world believe that opium posses therapeutic properties on many disorders, particularly diabetes mellitus (17, 18), in this study, we designed a diabetic animal model to verify this public believe on opium properties in an experimental manner.

## 2. Objectives

This study was carried out with the aim of assessing the effects of opium on number of peripheral blood cells and red blood cells (RBCs) indices in diabetic rats.

## 3. Materials and Methods

Opium was received by anti-drug section of Kerman Police (Iran). Based on their information, the origin of opium was Helmand, Afghanistan. GC-mass spectrometry analysis of this opium showed that it contained more than 30% alkaloids (from which morphine 16%, codeine 5.5%, thebaine 4.4%, papaverine 3.2% were the most abundant of them) and the rest consisted of non-alkaloidal organic and non-organic substances from which 13.5% was water (moisture). Streptozocin (STZ) was purchased from Pfizer Company (AG, Zurich, Switzerland). Glucose oxidase kit was also provided by Pars Azemoon Company (Tehran, Iran). All other materials were of analytical grade, obtained from standard sources.

### 3.1. Animals

In this experimental study, 28 male and 28 female Wistar rats (250-300 gram), were selected and divided into eight groups. Animals were housed on a 12 hour light-dark cycle and freely accessed to food and water. The study was approved by the Ethical Committee of the Rafsanjan University of Medical Sciences.

### 3.2. Induction of Diabetes and Addiction

All animals’ procedures were in accordance with “guide for the care and use of laboratory animals (NIH US publication No.85-23 revised 1985)”. Induction of diabetes and addiction was performed as described elsewhere (20). Briefly, equal number of animals (14 male and 14 female rats) were used for inducing diabetes by injection of streptozocin (dissolved in sodium citrate buffer, pH: 4.4) at a single dose of 60 mg/kg of the body weight intravenously into the tail vein. After three days, 0.5 mL of blood was obtained under ether anesthesia from orbit cavity by thin heparinized tube. The plasma level of glucose was measured using glucose oxidase method and animals with the glucose level of more than 250 mg/dL were regarded as diabetic. Seven diabetic male, seven diabetic female, seven non-diabetic male and seven non-diabetic female animals were treated with a double dose (8 AM and 8 PM) of opium for eight consecutive days by peritoneal injection (opium was dissolved in fresh saline). The control groups received merely normal saline as vehicle. The protocol of treatment was as follows: 30 mg/kg in the first day, 60 mg/kg in the second day, 90 mg/kg in the third day, 120 mg/kg in the fourth day, 150 mg/kg in the fifth day, and 150 mg/kg in the sixth day and the treatment continued as in the sixth day until eight days after treatment (150 mg/kg was the maximum tolerable dose for animals). The withdrawal in opium dependent rats was characterized by lacrimation, piloerection, salivation, an ejaculate like discharge, irritability, hyperactivity and wet-dog shakes. At the end of treatment protocol (9^th^ day) a single dose of 150 mg/kg opium was injected and three hours afterwards, animals were anesthetized with ether and then blood were obtained from orbit cavity by thin heparinized tube and was collected into heparinized coated tubes for complete blood count (CBC) analysis.

### 3.3. Blood Cells Counting

Total and differential leukocyte counts were carried out in samples obtained from peripheral blood. Total cell count was made in hemacytometer (T-890, Culter USA). Gimsa-stained blood films were used for differential white blood cell (WBC) counts.

### 3.4. Statistical analysis

Data were expressed as mean ± SD The results indicated that the distribution of the variable was normal. Data were analyzed using the student's t-test. A probability level of P < 0.05 was considered significant.

## 4. Results

### 4.1. The Effects of Opium Addiction on WBC Counts

The results of the effects of opium addiction on diabetic rats have been summarized in Table 1. The mean number of total WBC in opium-addicted diabetic female rats (7050 ± 280 cells/mm^3^) was significantly higher than what was observed in non opium-addicted diabetic female group (5537 ± 470 cells/mm^3^) (Table 1). Results of our study indicated that in both genders, the number of neutrophils of opium-addicted diabetic animals was obviously higher than the control group (2726 ± 99 and 1589 ± 155 cells/mm^3^ for males and, 4307 ± 300 and 2590 ± 98 cells/mm^3^ for females respectively), while the number of lymphocytes significantly decreases compared with control group (3184 ± 106 and 3811 ± 155 cells/mm^3^ for males, and 2742 ± 203 and 3045 ± 155 cells/mm^3^ for females respectively) (Table 1).

**Table 1:** Blood Parameters in Diabetic Addicted (Case) and Diabetic (Control) Male and Female Rats, Data From Seven Animals in Each Group

Blood Factor	Male, (Mean ± SD)		P Value	Female, (Mean ± SD)		P Value
	Case	Control		Case	Control	
**White blood cell, 10^3^/mm^3^**	5.928 ± 0.46	5.400 ± 0.35	0.383	7.050 ± 0.28	5.537 ± 0.47	0.018
**Neutrophil, 10^3^/mm^3^**	2.726 ± 0.099	1.589 ± 0.155	0.001	4.307 ± 0.299	2.590 ± 0.098	0.009
**Lymphocyte, 10^3^/mm^3^**	3.184 ± 0.107	3.811 ± 0.155	0.001	2.724 ± 0.203	3.045 ± 0.155	0.001
**Red blood cell, 10^6^/mm^3^**	7.834 ± 0.37	6.907 ± 0.42	0.128	5.077 ± 0.23	6.140 ± 0.15	0.002
**Hematocrit, %**	40.724 ± 1.93	36.771 ± 2.20	0.201	28.050 ± 1.10	36.075 ± 1.01	0.000
**Hemoglobin, g/dl**	13.314 ± 0.70	12.471 ± 0.67	0.406	9.500 ± 0.35	11.737 ± 0.26	0.000
**MCH,pg**	17.657 ± 0.39	17.000 ± 0.43	0.287	17.410 ± 0.21	16.725 ± 0.32	0.087
**MCV, µm^3^**	52.728 ± 1.12	52.185 ± 0.87	0.710	52.230 ± 0.77	53.500 ± 0.85	0.290
**MCHC, %**	33.514 ± 0.52	33.271 ± 0.38	0.717	33.430 ± 0.42	31.950 ± 0.60	0.067
**PLT , 10^3^/mm^3^**	1016.28 ± 76.70	1016.28 ± 76.70	0.732	1074.30 ± 82.86	981.12 ± 70.11	0.403

In opium-addicted diabetic female animals, the mean number of WBC (7050 ± 280 cells/mm^3^) and neutrophils (4307 ± 300 cells/mm^3^) significantly increased compared to diabetic addicted male group (5928 ± 460 and 2726 ± 99 cells/mm^3^ respectively), while the number of lymphocytes in diabetic addicted female (2742 ± 203 cells/mm^3^) decreased significantly compared with opium-addicted diabetic male group (3184 ± 106 cells/mm^3^) (Table 2).

**Table 2:** Comparison of Blood Parameters in Diabetic Addicted (Case) and Diabetic (Control) Male With Female Rats, Data From Seven Animals in Each Group

Blood Factor	Case, (Mean ± SD)		P Value	Control, (mean ± SD)		P Value
	Male	Female		Male	Female	
**White blood cell, 10^3^/mm^3^**	5.928 ± 0.46	7.050 ± 0.28	0.044	5.400 ± 0.35	5.537 ± 0.47	0.824
**Neutrophil, 10^3^/mm^3^**	2.726 ± 0.099	4.307 ± 0.300	0.007	1.590 ± 0.120	2.588 ± 0.098	0.000
**Lymphocyte, 10^3^/mm^3^**	3.184 ± 0.106	2.742 ± 0.203	0.001	3.810 ± 0.155	3.045 ± 0.155	0.002
**Red blood cell, 10^6^/mm^3^**	7.834 ± 0.37	5.077 ± 0.23	0.000	6.907 ± 0.42	6.140 ± 0.15	0.095
**Hematocrit, %**	40.742 ± 1.93	28.050 ± 1.10	0.000	36.771 ± 2.20	36.075 ± 1.01	0.769
**Hemoglobin, g/dl**	13.314 ± 0.70	9.500 ± 0.35	0.000	12.471 ± 0.67	11.737 ± 0.26	0.307
**MCH,pg**	17.657 ± 0.39	17.410 ± 0.21	0.561	17.000 ± 0.43	16.725 ± 0.32	0.623
**MCV, µm^3^**	52.728 ± 1.12	52.230 ± 0.77	0.722	52.185 ± 0.87	53.500 ± 0.85	0.303
**MCHC, %**	33.514 ± 0.52	33.430 ± 0.42	0.902	33.271 ± 0.38	33.950 ± 0.60	0.093
**PLT, 10^3^/mm^3^**	984.14 ± 49.62	1074.30 ± 82.86	0.367	1016.28 ± 76.70	981.12 ± 70.11	0.741

As shown in Table 3, for both genders, the number of neutrophils of opium-addicted animals was significantly higher than control (2612 ± 99 and 1370 ± 14 cells/mm^3^ for male rats and, 3916 ± 305 and 2735 ± 71 cells/mm^3^ for female rats, respectively), while the number of lymphocytes decreased when they were compared with control group (3198 ± 134 and 3529 ± 70 cells/mm^3^ for male, and 2842 ± 283 and 3477 ± 156 cells/mm^3^ for female respectively) (Table 3). Our results showed that in addicted female animals, the mean number of WBC (6850 ± 220 cells/mm^3^) and neutrophils (3916 ± 305 cells/mm^3^) increased significantly compared to opium-addicted male group (5850 ± 340 and 2612 ± 99 cells/mm^3^ respectively). While the number of lymphocytes in diabetic addicted female (2842 ± 283 cells/mm^3^) decreased significantly compared with opium-addicted diabetic male group (3198 ± 134 cells/mm^3^) (Table 4). No significant difference was observed between the mean number of total WBC, neutrophils and lymphocytes between opium-addicted diabetic and addicted non-diabetic male and female (Table 5), and diabetic and non-diabetic male and female rats (Table 6).

**Table 3:** Blood Factors in Addicted (Case) and Non-addicted (Control) Male and Female Rats, Data From Seven Animals in Each Group

Blood Factor	Male, (Mean ± SD)		P Value	Female, (Mean ± SD)		P Value
	Case	Control		Case	Control	
**White blood cell, 10^3^/mm^3^**	5.850 ± 0.34	5.066 ± 0.26	0.101	6.850 ± 0.22	6.100 ± 0.33	0.092
**Neutrophil, 10^3^/mm^3^**	2.612 ± 0.099	1.37 ± 0.014	0.000	3.916 ± 0.305	2.735 ± 0.071	0.023
**Lymphocyte, 10^3^/mm^3^**	3.198 ± 0.134	3.530 ± 0.070	0.000	2.842 ± 0.283	3.477 ± 0.156	0.010
**Red blood cell, 10^6^/mm^3^**	7.850 ± 0.34	6.516 ± 0.33	0.020	5.166 ± 0.28	5.883 ± 0.14	0.048
**Hematocrit, %**	40.666 ± 2.27	35.100 ± 1.79	0.084	28.450 ± 1.12	35.483 ± 0.69	0.000
**Hemoglobin, g/dl**	13.500 ± 0.56	12.116 ± 0.45	0.085	9.750 ± 0.38	11.750 ± 0.23	0.001
**MCH,pg**	17.400 ± 0.203	16.733 ± 0.363	0.141	17.100 ± 0.282	17.667 ± 0466	0.324
**MCV, µm^3^**	51.633 ± 0.842	51.800 ± 0.594	0.875	50.650 ± 0.337	54.683 ± 1.041	0.004
**MCHC, %**	33.733 ± 0.292	32.383 ± 0.659	0.091	33.833 ± 0.594	32.367 ± 0.711	0.145
**PLT, 10^3^/mm^3^**	1033.67 ± 50.019	913.17 ± 72.792	0.022	1296.83 ± 87.819	883.50 ± 40.642	0.002

**Table 4:** Comparison of Blood Parameters in Addicted (Case) and Non-addicted (Control) Male With Female Rats, Data From Seven Animals in Each Group

Blood Factor	Case, Mean ± SD		P Value	Control, Mean ± SD		P Value
	Male	Female		Male	Female	
**White blood cell, 10^3^/mm^3^**	5.850 ± 0.34	6.850 ± 0.22	0.036	5.066 ± 0.226	6.100 ± 0.33	0.035
**Neutrophil**	2.612 ± 0.099	3.916 ± 0.305	0.026	1.37 ± 0.014	2.735 ± 0.071	0.000
**Lymphocyte**	3.198 ± 0.134	2.842 ± 0.283	0.019	3.529 ± 0.070	3.477 ± 0.156	0.002
**Red blood cell, 10^6^/mm^3^**	7.850 ± 0.34	5.166 ± 0.28	0.000	6.516 ± 0.33	5.883 ± 0.14	0.117
**Hematocrit, %**	40.666 ± 2.27	28.450 ± 1.12	0.001	35.100 ± 1.79	35.483 ± 1.69	0.846
**Hemoglobin, g/dl**	13.500 ± 0.56	9.750 ± 0.38	0.000	12.116 ± 0.45	11.750 ± 0.23	0.491
**MCH,pg**	0.203 ± 17.400	0.282 ± 17.100	0.409	0.363 ± 16.733	0.466 ± 17.667	0.146
**MCV, µm^3^**	0.842 ± 51.633	0.337 ± 50.650	0.304	0.594 ± 51.800	1.041 ± 54.683	0.037
**MCHC, %**	0.292 ± 33.733	0.594 ± 33.833	0.883	0.659 ± 32.383	0.711 ± 32.367	0.987
**PLT, 10^3^/mm^3^**	50.01 ± 1033.6	87.8 ± 1296.8	0.026	72.792 ± 913.17	40.642 ± 883.50	0.731

**Table 5:** Blood Parameters in Diabetic Addicted and Non-diabetic Addicted Male and Female Rats, Data From Seven Animals in Each Group

Blood Factor	Male, Mean ± SD		P Value	Female, Mean ± SD		P Value
	Diabetic addicted	Addictednondiabetic		Diabetic addicted	Addicted non diabetic	
**White blood cell, 10^3^/mm^3^**	5.928 ± 0.46	5.850 ± 0.34	0.897	7.050 ± 0.28	6.850 ± 0.22	0.633
**Neutrophil**	2.726 ± 0.099	2.613 ± 0.099	0.590	4.307 ± 0.299	3.916 ± 0.305	0.556
**Lymphocyte**	3.184 ± 0.107	3.198 ± 0.134	0.747	2.742 ± 0.203	2.842 ± 0.283	0.605
**Red blood cell, 10^6^/mm^3^**	7.834 ± 0.37	7.850 ± 0.34	0.976	5.077 ± 0.23	5.166 ± 0.28	0.813
**Hemoglobin, g/dl**	13.314 ± 0.70	13.500 ± 0.56	0.844	9.500 ± 0.35	9.750 ± 0.38	0.656
**Hematocrit, %**	40.742 ± 1.93	40.666 ± 2.27	0.980	28.050 ± 1.10	28.450 ± 1.12	0.518
**MCH,pg**	17.657 ± 0.39	17.400 ± 0.20	0.453	17.410 ± 0.21	17.100 ± 0.28	0.394
**MCV, µm^3^**	52.72 ± 1.12	51.633 ± 0.84	0.577	52.230 ± 0.77	50.650 ± 0.33	0.088
**MCHC, %**	33.514 ± 0.52	33.733 ± 0.29	0.725	33.430 ± 0.42	33.833 ± 0.59	0.581
**PLT, 10^3^/mm^3^**	984.14 ± 49.62	1033.66 ± 50.01	0.497	1074.30 ± 82.86	1296.83 ± 87.81	0.089

**Table 6:** Blood Factors in Diabetic and Non-diabetic Male and Female Rats, Data From Seven Animals in Each Group

	Male, Mean ± SD		P Value	Female, Mean ± SD		P Value
	Diabetic	Non diabetic		Diabetic	Non diabetic	
**White blood cell, 10^3^/mm^3^**	5.400 ± 0.35	5.066 ± 0.26	0.483	5.537 ± 0.47	6.100 ± 0.33	0.382
**Neutrophil**	1.589 ± 0.155	1.367 ± 0.144	0.564	1.250 ± 0.098	2.735 ± 0.071	0.422
**Lymphocyte**	3.811 ± 0.155	3.529 ± 0.093	0.804	3.045 ± 0.155	3.477 ± 0.156	0.621
**Red blood cell, 10^6^/mm^3^**	6.907 ± 0.42	5.516 ± 0.33	0.496	6.140 ± 0.15	5.883 ± 0.14	0.259
**Hemoglobin, g/dl**	12.471 ± 0.67	12.116 ± 0.45	0.683	11.737 ± 0.26	11.750 ± 0.23	0.973
**Hematocrit, %**	36.771 ± 2.20	35.100 ± 1.79	0.577	36.075 ± 1.01	35.483 ± 0.69	0.662
**MCH,pg**	17.000 ± 0.43	16.733 ± 0.36	0.648	16.725 ± 0.32	17.666 ± 0.46	0.131
**MCV, µm^3^**	52.185 ± 0.87	51.800 ± 0.59	0.723	53.500 ± 0.85	54.683 ± 1.04	0.394
**MCHC, %**	33.271 ± 0.38	32.383 ± 0.65	0.278	31.950 ± 0.60	32.366 ± 0.71	0.665
**PLT, 10^3^/mm^3^**	1016.28 ± 76.70	913.16 ± 72.79	0.350	981.12 ± 70.11	883.50 ± 40.64	0.254

### 4.2. The Effects of Opium Addiction on RBC Counts and RBC-Related Indices

In opium-addicted diabetic male group, the mean number of RBC increased significantly as compared with diabetic male group [(7.834 ± 0.370) × 10^6^ cell/mm^3^ versus (6.907 ± 0.370) × 10^6^ cell/mm^3^]. However, in opium-addicted diabetic female rats, the mean number of RBC decreased significantly compared to diabetic non-addicted female group [(5.077 ± 0.230) × 10^6^ cell/mm^3^ versus (6140 ± 150) × 10^6^ cell/mm^3^ ] (Table 1).

As demonstrated in *Table 2*, for diabetic-addicted female animals, the mean number of RBC [(5.077 ± 0.230) × 10^6^ cell/mm^3^] and the mean levels of RBC-related indices such as hematocrit and hemoglobin decreased significantly compared to diabetic-addicted male group [(7.834 ± 0.370) × 10^6^ cell/mm^3^] (Table 2).

The results showed that in opium-addicted male group the mean number of RBC and RBC indices increased significantly as compared with non-addicted male group [(7.850 ± 0.34) × 10^6^ cell/mm^3^ versus (6.516 ± 0.33) × 10^6^ cell/mm^3^]. Whereas, in opium-addicted female rats the mean counts of RBC and RBC indices decreased in comparison with non-addicted females [(5.166 ± 0.28) × 10^6^ cell/mm^3^ versus (5.883 ± 0.14) × 10^6^ cell/mm^3^] (Table 3).

We found that in opium-addicted female animals the mean number of RBC [(5.166 ± 0.28) × 10^6^ cell/mm^3^] and the mean levels of RBC-related indices such as hematocrit and hemoglobin decreased significantly compared to diabetic-addicted male group [(7.850 ± 0.34) × 10^6^ cell/mm^3^] (Table 4). No significant difference was found in RBC and RBC indices between addicted-diabetic and addicted non-diabetic male and female (Table 5), and diabetic and non-diabetic male and female rats (Table 6).

### 4.3. The effects of opium addiction on platelet counts

In both male and female rats, the mean number of platelets in opium-addicted diabetic rats was significantly lower than those observed in addicted non-diabetic groups (Table 5). However, no significant differences were found between other groups (P > 0.05).

## 5. Discussion

Opium abuse and its derivatives are still a worldwide problem and a huge amount of currencies are spent annually for these dangerous latent materials. Due to the importance of the side effects of the opium on several systems and organs of the body (17-20) and to give awareness and information to the society, we aimed this project at the effects of opium on hematological parameters. Furthermore, because our country is located on the traffic band of opium smuggling to the European courtiers, it is essential to inform the public of the unwanted effects of opium on health.

In addition to several social and economical difficulties raised from narcotic drugs, the results of the present study showed the profound effects of opium on the total and differential counts of peripheral WBC, the number of RBC and the levels of some RBC-related parameters. There was a remarkable difference between male and female groups regarding the total and differential counts of WBC in opium addicted and non-addicted animals. In this study, we showed that opium addiction has a profound influence on the mean total WBC count. We have observed that the mean number of total WBC in addicted-diabetic female rats was significantly higher than the one observed in non-addicted diabetic female group. Whereas, in male rats, although the mean counts of total WBC in opium-addicted diabetic group was higher than non-addicted diabetic group, but the difference was not significant. The precise mechanisms by which opium addiction leads to an elevation of WBC levels and alterations in the differential WBC counts are yet to be determined. The differentiation of leukocytes from bone marrow stem cells, inflammation, infections and modification of the expression of adhesion molecules on the endothelial cells can influence the number of peripheral WBC. It has been reported that addiction suppresses the immune system and the addicted individuals are more susceptible to infectious diseases (21). Accordingly, some effects of opium addiction on the number of WBC may be mediated through infection and inflammation. Morphine, a component of opium, induces the release of catecholamines which is known to increase the leukocyte count (22). Elevations in the peripheral blood leukocyte count may be induced by direct injury of epithelial and endothelial surfaces and/or changes in cytokine levels (in particular IL-6) caused by components of opium (23). In agreement with these findings, Asakura *et al*. demonstrated that the risk of acute infection is higher in heroin intravenous drug addicted patients (24). It should be noted that the hemopoises is regulated by a complex network of cytokines such as colony stimulating factors. The opium or some of its derivative may interfere with cytokine network and in turn influence the production of WBC. It has been demonstrated that opium or some of its derivatives can affect the secretion of cytokines such as IL-2, IL-4, IL-5, IL-10, IFN-γ and TGF-β (19, 24). In the present study in both male and female rats, the number of lymphocytes in addicted-diabetic groups was significantly lower than that observed in diabetic non-addicted groups. Reduced number of lymphocytes has also been reported in addicted dogs (25). These observations represent that the opium addiction have profound adverse effects on lymphocytes counts. These effects may be attributed to the apoptotic properties of opium or its derivatives on lymphocytes. Indeed, the apoptotic effects of morphine and heroin on lymphocytes and other cells have been demonstrated by some investigators (26, 27). Moreover, opium or some of its derivative may reduce lymphopoises via interference with cytokines, which are responsible for lymphocytes differentiation from bone marrow stem cells.

The results of the present study showed that in diabetic opium-addicted male group, the counts of RBC increased significantly as compared with diabetic male group, while in diabetic opium-addicted female rats, the mean number of RBC decreased significantly in comparison with non-addicted diabetic female group. These differences may be attributed to differential effects of sex hormones on RBC-related parameters, so that opium or some of its derivatives may influence the number of RBC and RBC-related parameters in coordination with sex hormones. In a previous study, we showed that some other parameters are gender dependent in addicted subjects (17-19). However, anemia has been reported as one of the prominent clinical features of addiction (28). This disorder is also clearly seen in our study. According to the obtained results of this investigation, the RBC indices are markedly decreased, in fact, it probably means that opium-addicts are anemic, and more surprisingly this anemia is more remarkably obvious in female addicted-diabetic animals. Regarding the data base information in literature, it could perhaps be due to the direct and/or indirect impact of morphine on bone marrow stem cells and RBC progenitor cells and these results are consistent with the results of Govitrapong et al. (29). However, further studies are needed to obtain a comprehensive understanding of the mechanisms involved in these alterations. In both genders, the counts of platelets in opium-addicted diabetic rats were significantly lower than those observed in diabetic non-addicted groups. The precise mechanisms involved for these observations remain to be determined. There was a sharp difference between male and female WBC counts of addicted and non-addicted animals. In addicted groups, the number of neutrophils was higher than the control group, probably due to the imbalances effect of opium on the immune system.

Also, it could probably be concluded that in the opium addicts the risk of infection enhances due to the weakness of immune system as a result of opium. In case of lymphocytes, as it could be predicted the lymphocytes count in both genders in diabetic addicted decreases. This in away confirms the risk of acute infection in these animals. Based on the results of our study and the findings of similar studies, opium may have a suppressive influence on the bone marrow including stem cells and RBC progenitor cells. In addition, herein we showed that female opium-addicted diabetic animals were more susceptible to anemic than males.

It is worthy to note that some patients, in particular diabetics’ individuals often consume opium at high doses for a long period of time (17) that could be deleterious for the patients' blood cells and their immune system.
